# Accuracy and Confidence of Visual Short-Term Memory Do Not Go Hand-In-Hand: Behavioral and Neural Dissociations

**DOI:** 10.1371/journal.pone.0090808

**Published:** 2014-03-24

**Authors:** Silvia Bona, Juha Silvanto

**Affiliations:** 1 Brain Research Unit, O.V. Lounasmaa Laboratory, School of Science, Aalto University, Espoo, Finland; 2 BioMag Laboratory, HUS Medical Imaging Center, Helsinki University Central Hospital, Helsinki, Finland; 3 Department of Behavioural Sciences, University of Helsinki, Helsinki, Finland; 4 Department of Psychology, Faculty of Science and Technology, University of Westminster, London, United Kingdom; University of Tokyo, Japan

## Abstract

Currently influential models of working memory posit that memory content is highly accessible to conscious inspection. These models predict that metacognition of memory performance should go hand-in-hand with the accuracy of the underlying memory representation. To test this view, we investigated how visual information presented during the maintenance period affects VSTM accuracy and confidence. We used a delayed cue–target orientation discrimination task in which participants were asked to hold in memory a grating, and during the maintenance period a second memory cue could be presented. VSTM accuracy of the first memory cue was impaired when the orientation of the second memory cue was sufficiently different. However, participants' response confidence was reduced whenever the second memory cue was presented; thus VSTM accuracy and confidence were dissociated. In a second experiment, we applied transcranial direct current stimulation (tDCS) over the right dorsolateral prefrontal cortex (DLPFC) to investigate the causal role of this region in VSTM metacognition. Relative to the sham condition, anodal tDCS induced a general reduction in confidence ratings but did not affect VSTM accuracy. Overall, these results indicate that our metacognition of memory performance is influenced by factors other than the accuracy of the underlying memory representation.

## Introduction

Metacognition refers to insight into one's own cognitive experiences and processes [1], [2]; in memory research, this knowledge is referred to as metamemory. From a theoretical perspective, memory processes can be separated into two different levels: an “object” level (reflecting the actual memory trace, on which objective memory performance is based) and a “meta” level (containing an imperfect model of the object level) which can monitor and modify processes occurring at the object level [3], [4]. The meta-level functions are commonly assessed with the use of confidence ratings, which are participants' subjective assessments of their performance in memory tasks [4], [5], [6], [7].

Confidence ratings have been shown to positively correlate with the strength of the underlying memory trace [5], [8], [9], [10] and they can be a good predictor of memory accuracy [11], [12], [13]. This indicates that confidence judgments and accuracy are based on the same underlying representation [5], and according to the *trace access theory* [14], [15], [16] a direct access to the contents of memory is available when confidence and recognition judgments are made. However, there is evidence to indicate that objective and subjective aspects (i.e. “object” level and “meta” level) of memory can be dissociated [9], [17], [18], [19], suggesting that they may not be based entirely on the same source of information. For example, it has been shown that the ease of retrieval contributes to retrospective confidence judgments independently of accuracy [20], indicating that partly different variables affect confidence and accuracy dimensions [5], [21], as postulated by the *accessibility hypothesis* [22].

Although the dissociation between objective memory performance and its metacognitive and introspective aspects has been widely studied in the context of long-term memory, so far this issue has received very little interest in the study of working memory/visual short-term memory. The issue is theoretically important however; current models of working memory posit that memory contents are immediately accessible to consciousness [23], [24], and a prediction that follows from this is that subjective evaluations of memory performance should closely reflect the accuracy of the underlying memory representation (on which measures of accuracy are based). The existing evidence for this view is inconsistent. In a recent study by Rademaker et al. [12], confidence ratings strongly predicted the likelihood that the cued grating was successfully maintained, consistent with the view that working memory content are robustly available to conscious experience. In contrast, another study observed a double dissociation between VSTM accuracy and the introspection of VSTM content [25]. Specifically, the features and visibility of distracters presented during the maintenance period differentially affected the objective and subjective measures of VSTM, indicating that the subjective experience may not always accurately reflect the underlying VSTM representation. However, Bona et al. [25] assessed memory vividness rather than confidence ratings, and thus it did not directly assess participants' insight into their memory performance.

Here we investigated metacognition of visual short-term memory by assessing whether confidence ratings and VSTM accuracy are dissociable at the behavioral and cortical level. In Experiment 1, we assessed how visual information presented during the maintenance period (which either needs to be encoded into VSTM or merely passively observed) affects these measures, by using a delayed cue-target orientation discrimination task (as previously used by Bona et al. [25]) and Silvanto and Soto [26]). Accuracy and confidence were assessed on a trial-by-trial basis. If confidence and accuracy are based on the same source of information, as predicted by the *trace access theory* [14], [15], [16], the visual stimuli presented during the delay period should affect confidence and accuracy in the same manner; VSTM and confidence should go hand-in-hand. In contrast, if accuracy and confidence are based on partly different sources of information then we might see circumstances in which our manipulations would differentially affect VSTM accuracy and confidence.

**Figure 1:**
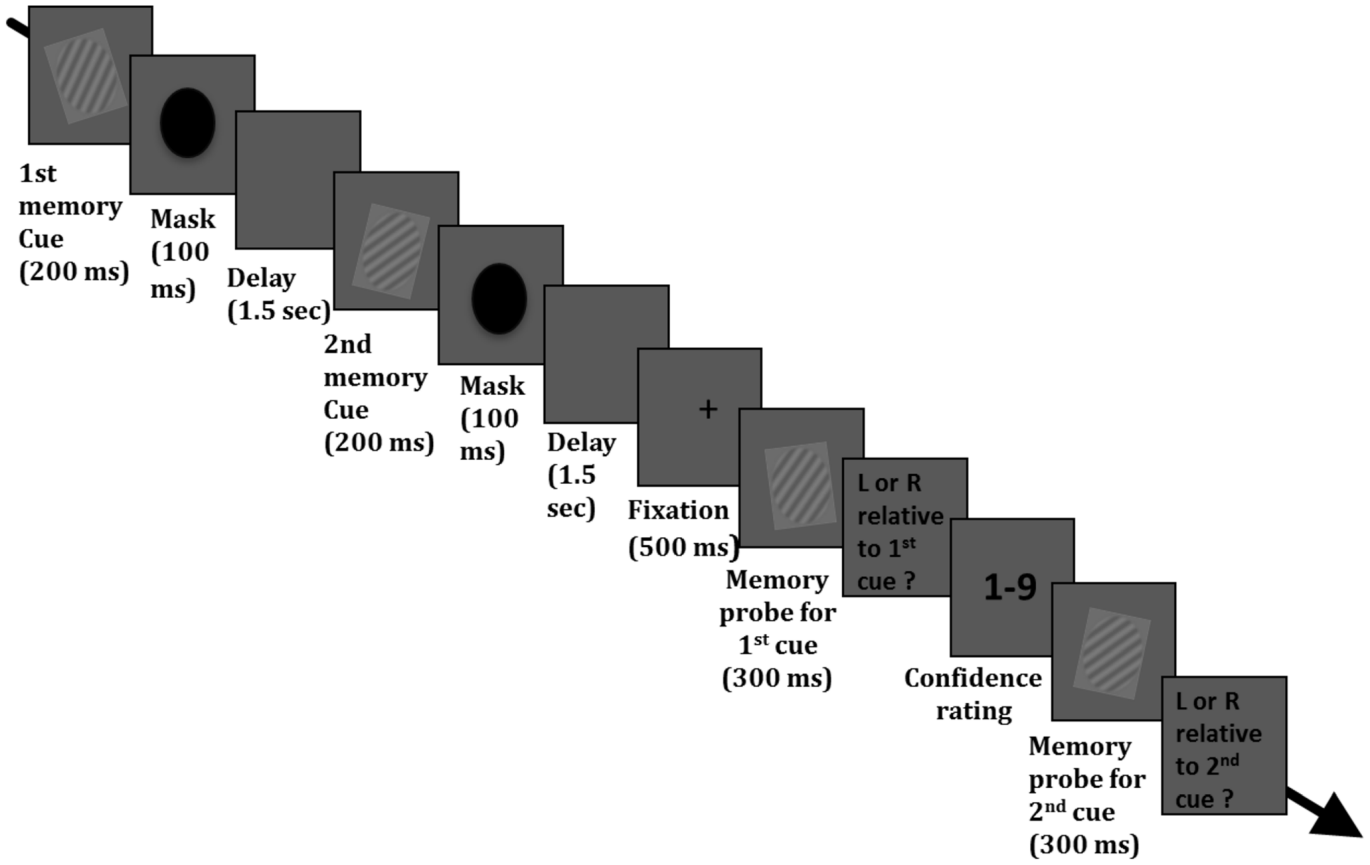
Timeline of an experimental trial. Participants were asked to maintain in memory the orientation of a memory cue (grating); at the end of each trial they were asked to indicate whether a test probe was tilted to the left or to the right relative to the memory cue. In addition, participants provided a confidence rating for this memory jdugment by using a scale from 1–9 (1 = not confident at all; 9 = extremely confident). On 75% of the trials, the first memory cue was followed by a second cue; this could be either identical to the first cue, or its orientation differed by 10 or 40 degrees. In the *active condition*, participants were asked to hold its orientation in memory. Thus in this condition, participants were required to hold the orientation of two cues on each trial. The maintenance of the 2^nd^ cue was assessed in the same manner as that of the 1^st^ cue: they were asked to indicate whether a test probe was tilted to the left or to the right relative to the memory cue. The memory judgment relating to the 2^nd^ cue was always made after the two responses (accuracy and confidence responses) relating to the first cue. In the *passive condition*, participants were not required to hold the 2^nd^ cue in memory. To ensure that they attended to the 2^nd^ cue, participants were asked to indicate at the end of the trial whether or not the 2^nd^ cue was presented.

On each trial, participants were presented with two gratings appearing in a sequence: in the *active* condition, both gratings needed to be held in memory and VSTM accuracy was assessed separately for both at the end of the trial (see Figure 1). The VSTM task required participants to judge, for each memory cue, whether the test probes were tilted to the left or to the right relative to the memory cues. This task required an explicit comparison between the orientation of the test stimuli and the memory cues (which cannot be performed by mere familiarity/recognition as the orientations of the probes and the memory cues were never the same). In the *passive* condition, the second memory cue was passively viewed and not held in memory. This passive condition was included to determine whether any effects found in the active condition is due to an increase in memory load or induced by the mere presentation of a distracter. We predicted that objective VSTM accuracy ought to be impaired when the orientations of the two stimuli differ sufficiently, according to our previous studies using the same paradigm [25], [26] and consistent with the phenomenon of competition between orientation-selective channels, the width of which is believed to be in the range of 30–40 deg [27], [28], [29]. The key question is whether confidence ratings are affected by the second stimulus in the same manner.

In a second experiment, using the paradigm developed in Experiment 1, we examined the neural basis of VSTM metacognition by the use of transcranial direct current stimulation (tDCS).

A number of brain areas have been implicated in VSTM. One such region is the posterior parietal cortex (PPC) [30], [31], [32] especially in the right hemisphere [33], [34], [35]. A second region strongly implicated in VSTM is the prefrontal cortex; several neuroimaging studies have shown an increase in neuronal activity in particular in its dorsolateral region (BA 46 and 9) during working memory tasks [36], [37], [38], [39], [40], [41]. In the present study we focused on the prefrontal cortex, as it is most commonly implicated not only in VSTM but also in metacognitive abilities [e.g 42,43,44,45,46], especially its dorsolateral portion [21], [47]. For example, Henson et al. [47] found enhanced activity in the right dorsolateral prefrontal cortex for low-confidence (compared to high-confidence) judgments, interpreting this activation pattern as reflecting an increased involvement of this area in situations likely to require more monitoring of the retrieved information. Furthermore, patients suffering from dorsolateral prefrontal damages perform worse than controls in tasks requiring confidence judgments [48], [49]. The objective of Experiment 2 was to investigate the *causal* role of this region in metamemory by the use of tDCS, which is a noninvasive brain stimulation technique that allows to modulate the spontaneous cortical activity in the brain [50], [51], [52]. The effects of the stimulation depend on the polarity of the current flow: anodal tDCS is assumed to increase the brain excitability of the underlying region whereas cathodal tDCS generally leads to a decrease in the excitability [52], [53], [54], [55]. While the effects of anodal tDCS are relatively well established in the literature [e.g. 52,56,57,58], the effects of cathodal polarization are more controversial [52], [55], [59], [60], [61], [62], [63], [64]. Therefore we choose to rely on an anodal stimulation experimental design, aiming to increase the excitability of DLPFC in order to investigate the role of this region in metacognition of VSTM. The choice of anodal protocol was also motivated by several previous studies having successfully modulated WM performance by applying anodal tDCS over dorsolateral prefrontal cortex [65], [66], [67], [68].

## Materials and Methods

### Experiment 1

#### Subjects

Thirty-two students from University of Helsinki with normal or corrected-to-normal vision took part in the study. Sixteen participants (7 males, mean age = 23.9; SD: 1.71) performed the active condition of the study and the remaining sixteen (8 males, mean age = 24.6; SD: 2.18) performed the passive condition (see “stimuli and experimental procedure” section). All participants were naïve to the aim of the study and provided written informed consent. The study was performed in agreement with the Declaration of Helsinki and approved by the ethics committee of the Hospital District of Helsinki and Uusimaa.

#### Stimuli and experimental procedure

Figure 1 shows an example of an experimental trial. Participants were seated at a viewing distance of 57 cm from the screen and stimuli were presented on a 19-inch monitor (1280×1024) with a refresh rate of 60 Hz. The experiment was controlled by E-prime v2.0. The task required the maintenance of a sinusoidal luminance-modulated grating (as previously used by Bona et al. [25], Silvanto ad Soto [26]), Magnussen et al. [69]; Magnussen and Greenlee [70]. Each trial began with a black fixation cross appearing in the middle of the screen for 1000 ms, followed by a blank screen for 500 ms. Participants were then presented with a memory cue, so-called “1^st^ memory cue” (orientation 10, 20, 30, 40 or 50 deg. to the left or right from vertical; 0.1 Michelson contrast; spatial frequency 1 cycle/degree; diameter 4 degrees of visual angle from a viewing distance of 57 cm) appearing on the screen for 200 msec and followed by a 100 ms duration mask (a black circle covering the entire area of the previous grating) in order to reduce any after-image effect. On 75% of trials, after a 1.5 sec delay, a second memory cue was presented; this was either identical to the first cue, or its orientation differed by 10 or 40 degrees. This second cue was presented for 200 ms and followed by a 100 msec duration mask. Spatial frequency, contrast, size and location were the same as those of the first memory cue. On 25% of trials, the second memory cue was not presented, in order to obtain a baseline level of performance for the first memory cue. Participants in the *active* condition were instructed to hold the orientation of the second memory cue in memory; participants in the *passive* condition were not required to do so. At the end of the maintenance period, a memory test probe (tilted 10° either to the left or right relative to the first memory cue) was presented for 300 ms and participants had to indicate with a button press (during an unlimited time window) whether the test probe was tilted to the left or to the right relative to the first memory cue. The test probe and the first memory cue were always tilted to the same direction (i.e both tilted to the right or both tilted to the left) and their orientation difference was always 10 deg. After this response, confidence rating for the 1^st^ memory judgment was given on a scale from 1-9 (1 = not confident at all; 9 = extremely confident). Finally, to ensure that participants in the active condition were holding in memory the second memory cue, its maintenance was assessed in the same manner as the first memory cue: specifically, a second test probe was presented (tilted 10 deg. either to the left or to the right relative to the second memory cue) and participants had to indicate the direction of the tilt relative to the second memory cue.

As mentioned in the Introduction, the passive condition was included to investigate whether any effects found in the active condition is due to an increase of memory load (as in the active condition participants are required to maintain in memory also the second cue), or whether such effects are induced by the mere passive viewing of distracting information. Confidence ratings were not collected for the discrimination task relating to the second cue in order to avoid confusion that might arise between the memory judgments of first and second memory cue. In the passive condition, to ensure that they attended the second cue, participants were asked to indicate at the end of the trial (after they had given responses relating to the 1^st^ memory cue) whether or not the second cue was presented. In both conditions participants performed 6 blocks, each one containing 80 trials.

### Experiment 2

#### Subjects

Fifteen healthy students from University of Helsinki (7 males, mean age = 25.13; SD: 3.76) with normal or corrected-to-normal vision took part in the study. None of them had participated in Experiment 1. Participants were naïve as to the aims of the study and provided informed consent. Furthermore, a screening was carried out with all participants, in order to exclude history of epilepsy as well as neurologic, psychiatric and cardiac diseases. The study was performed in agreement with the Declaration of Helsinki and approved by the ethics committee of the Hospital District of Helsinki and Uusimaa.

#### Transcranial direct current stimulation

Transcranial direct current stimulation was delivered by using a battery-driven constant current stimulator (Eldith, Neuroconn, Ilmenau, Germany) through a pair of 7×5 cm sponge electrodes embedded in a saline-soaked solution. Current was applied for 20 minutes at a 2 mA constant intensity, according to safety parameters proposed for healthy participants [71]. Previous studies have shown that these parameters effectively modulate cortical excitability [57], [72], [73]. Current density (0.057 mA/cm2) was maintained below the safety limits [74] for the entire duration of the stimulation. Anodal (so-called active) electrode was placed over right DLPFC, corresponding to F4, according to the International 10–20 EEG system [66], [75] while the cathodal, so-called reference electrode was placed over the contralateral supraorbital area (see e.g [65], [66], [75] for previous studies using this montage). Electrodes were fixed in place by using elastic bands. All participants performed two different stimulation sessions (anodal and sham stimulation) with an interval ranging from two to six days, in order to minimize any carry-over effects. For sham stimulation the electrodes were placed in the same position as in the anodal stimulation but current was slowly turned off after 10 seconds; this procedure has been shown to diminish sensory differences between anodal and sham stimulation [55]. Both anodal and sham sessions lasted for 20 minutes. The order of sham and anodal stimulation was counterbalanced across participants, so that half of the participants began with sham condition and the remaining half with the anodal condition. None of the participants reported sensory differences between the anodal and sham sessions.

#### Stimuli and experimental procedure

Participants were seated at a viewing distance of 57 cm from the screen and stimuli were presented on a 19-inch monitor (1280×1024) with a refresh rate of 60 Hz. Stimuli and task were identical to Experiment 1. As the effect of the second cue in our paradigm did not differ depending on whether it needed to be held in memory or passively viewed (see results below), we included in Experiment 2 only the active condition. In both anodal and sham sessions, participants performed two blocks of the VTSM task before the stimulation (i.e. pre-tDCS condition) and two blocks immediately following the stimulation (post-tDCS conditions). Twenty minutes of stimulation at 2 mA are expected to induce effects covering approximately 10 minutes duration [57], which was approximately the duration needed to complete the two blocks.

## Results

### Experiment 1

**Figure 2:**
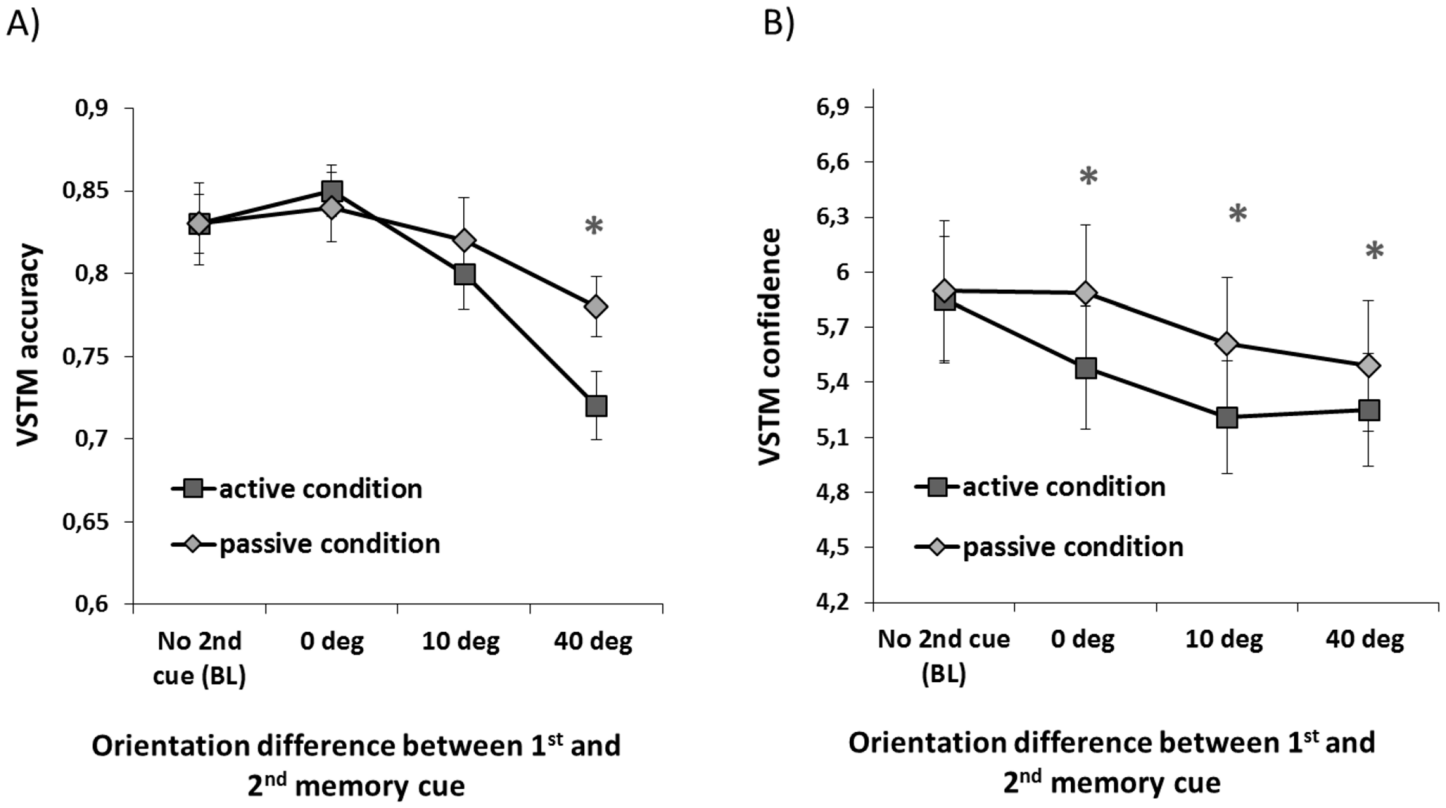
Dissociation between VSTM accuracy and confidence in Experiment 1. **A**) VSTM accuracy for the 1^st^ memory cue as a function of orientation difference between 1^st^ and 2^nd^ memory cue in the active and passive conditions. Relative to the baseline condition (i.e. when no 2^nd^ cue was presented) memory accuracy was reduced only when orientation difference between the two cues was 40 degrees; this effect was not significantly modulated by memory load (although a trend for an interaction between memory load and orientation was present). The asterisks indicate conditions which significantly differ from the BL condition. Error bars indicate ±1 SEM. **B**) VSTM confidence as a function of orientation difference between the 1^st^ and 2^nd^ memory cue in active and passive condition. Relative to the baseline condition (i.e. when no 2^nd^ cue was presented) confidence ratings were significantly reduced whenever the 2nd cue was presented; this effect was not significantly modulated by the memory load or by the orientation of the 2nd cue. The asterisks indicate conditions which significantly differ from the BL condition. Error bars indicate ±1 SEM.

The aim of Experiment 1 was to investigate whether the introduction of visual information (“2^nd^ memory cue”) during the maintenance of orientation information has the same impact on VSTM accuracy and confidence. Furthermore, we aimed to assess whether any such effect arise when the 2^nd^ cue needs to be encoded into VSTM or is merely passively observed. To this purpose we carried an ANOVA with “trial type” (baseline; i.e. no 2nd cue), 0 deg difference between 1^st^ and 2n cue, 10 deg difference, 40 deg difference) as a within-subjects factor and “memory load” (active condition, passive condition) as a between-subjects factor. The results for accuracy and confidence are shown in Figure 2.

#### The impact of the 2nd cue and memory load on VSTM accuracy

The ANOVA on accuracy revealed a significant main effect of trial type (F(3,90) = 21.1; p<.001; partial η^2^ = .41), no main effect of load (F(3,90) = .43; p = .52; partial η^2^ = .09) and a nonsignificant trend in the interaction between trial type and memory load (F(3,90) = 2.14; p = .11; partial η^2^ = .07). Further analysis on the effect of trial type showed that, relative to the baseline condition (i.e. when the second cue was not presented), memory accuracy was reduced when the second memory cue differed from the first one by 40 degrees (t(31) = 5.6; p<.001) but not when they were identical (t(31) = 1.3; p = .20) or differed by 10 degrees (t(31) = 1.8; p = .09).

#### The impact of the 2nd cue and memory load on Confidence ratings

The ANOVA on confidence revealed a significant main effect of trial type (F(3,90) = 12.3; p<.001; partial η^2^ = .29), no main effect of memory load (F(3,90) = .33; p = .57; partial η^2^ = .09) and no significant interaction between trial type and memory load (F(3,90) = 1.5; p = .21; partial η^2^ = .05). Further analysis on the effect of trial type showed that confidence rating was reduced (relative to the baseline condition) whenever the second cue was presented (*0 deg vs. baseline*: t(31) = 2.1; p = .04; *10 deg vs baseline*: t(31) = 4.9; p<.001; *40 deg vs baseline*: t(31) = 5.2; p<.001).

Thus VSTM accuracy and response confidence were differentially affected by the presentation of the second memory cue: VSTM accuracy of the first memory cue was impaired when the orientation of the second cue was sufficiently different from the first memory item. In contrast, response confidence of the first memory cue was reduced whenever the second cue was presented. These effects were not modulated by memory load.

### Experiment 2

**Figure 3:**
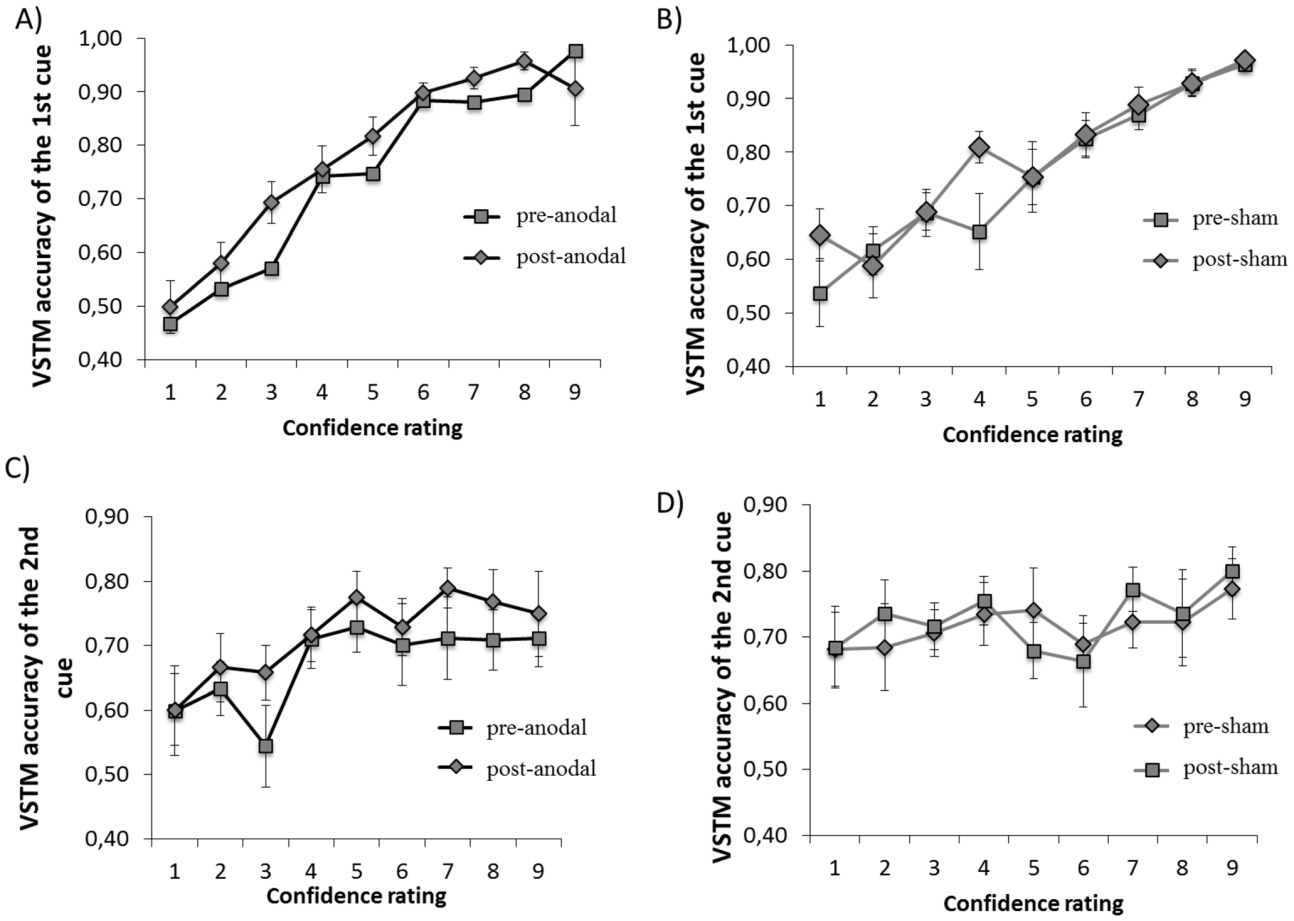
Correlation between VSTM accuracy and confidence for each tDCS condition. Correlation between confidence ratings and VSTM accuracy of the 1^st^ memory cue in anodal-tDCS conditions (panel A) and sham-tDCS conditions (panel B). Correlation between confidence ratings of 1^st^ memory cue and VSTM accuracy of the 2^st^ memory cue in anodal-tDCS conditions (Panel C) and sham-tDCS conditions (Panel D) Error bras indicate ±1 SEM.

The aim of Experiment 2 was to investigate the role of the right dorsolateral prefrontal cortex in VSTM metacognition. For this experiment, we used only the active condition from Experiment 1. In order to obtain an overall measure of metacognition independently of the similarity between first and second memory cue, we first assessed the overall relationship between the VSTM accuracy of the first memory cue and its confidence ratings for each tDCS condition (see Figure 3). A statistically significant correlation was found in all conditions; this correlation was very similar in all the tDCS conditions (*pre-sham*: r = .61; p<.01; *post-sham*: r = .61; p<.01; *pre-anodal*: r = .67; p<.01; *post-anodal*: r = .68; p<.01). Thus tDCS did not induce a general modulation in the correlation between VSTM accuracy and response confidence (i.e. the slope of the psychometric function in Figure 3). Figure 3A does however suggest a leftward shift in the psychometric function from pre-anodal tDCS condition to post-anodal tDCS condition, a shift not present in the sham condition. This indicates that each level of confidence rating was associated with a higher level of VSTM accuracy after anodal tDCS, indicative of a bias shift towards more conservative confidence ratings. (The impact of tDCS on VSTM accuracy and confidence as a function of stimulus condition is investigated statistically in the next section).

#### The impact of tDCS on VSTM accuracy

**Figure 4:**
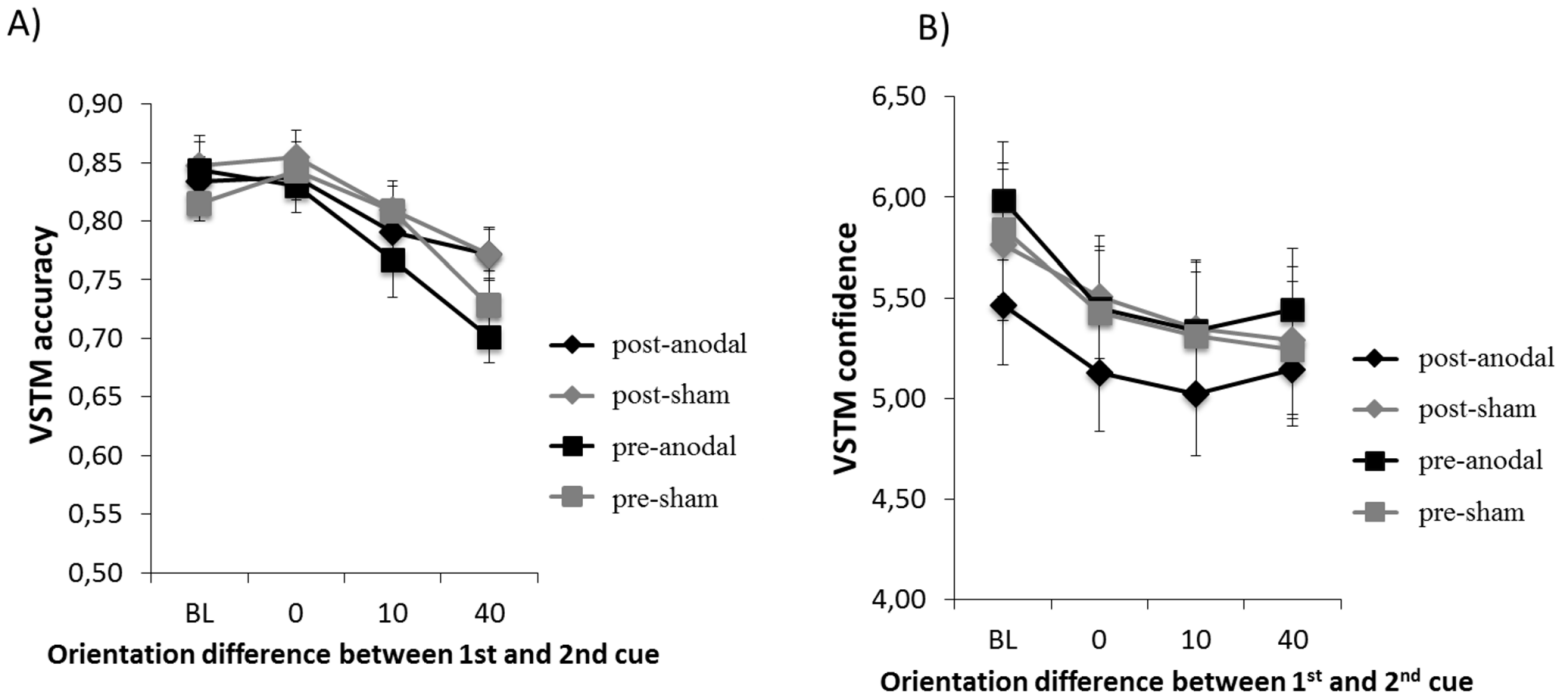
Dissociation between VSTM accuracy and confidence in Experiment 2: differential effects of tDCS and orientation similarity between the memory cues. (**A**) Mean (n = 15) VSTM accuracy as a function of stimulus condition for each tDCS condition. A significant main effect of stimulus condition was found, with accuracy being reduced when orientation difference between first and second memory cue was 10 or 40 deg, with largest effect found at 40 degrees. In addition a main effect of session order was found (higher performance in post-tDCS versus pre-tDCS), suggesting a slight learning effect. No other main effect or interaction was observed. Error bars indicate ±1 SEM. A similar pattern of results was observed also in reaction times analysis. (**B**) Mean (n = 15) confidence ratings as a function of stimulus condition for each tDCS condition. A significant interaction between tDCS condition and session order was observed, such that confidence ratings were generally lower in the post-real tDCS session. As in Experiment 1, confidence ratings were reduced whenever the 2^nd^ cue was presented.

We then analysed the results as a function of the orientation similarity between the first and the second memory cue (as done in Experiment 1). The impact of tDCS on VSTM accuracy as a function of stimulus condition is shown in Figure 4A. An ANOVA with stimulus condition (BL, 0 deg difference, 10 deg difference, 40 deg difference), tDCS condition (anodal or sham) and session order (pre or post) as main factors was carried out. A main effect of stimulus condition was significant (F(3,42) = 14.92; p<.001; partial η^2^ = .52). Post-hoc comparisons revealed that, relative to the baseline condition (i.e. when the second cue was not presented) memory accuracy was reduced when the second memory cue differed from the first one by 40 degrees (t(14) = 4.9; p<0.001) and 10 degrees (t(14 = 3.4; p = 0.004) but not when they were identical (t(14) = .58; p = .57). Furthermore, performance was significantly worse when the orientation difference was 40 deg than 10 deg (t(14) = 3.04; p = 0.009). A main effect of session order was also observed, with performance higher post versus pre-tDCS (F(1,14) = 6.37; p = .024; partial η^2^ = .31), indicating a slight learning effect. No other main effect or interaction was significant. The lack of main effect or interactions involving the tDCS condition (anodal versus sham) indicates that tDCS did not modulate VSTM accuracy.

We also analysed the impact of tDCS on reaction times. An ANOVA with tDCS condition (anodal or sham) and session order (pre or post) as main factors was performed.

A significant main effect of session order was observed (F(14) = 19.16; p = .001; partial η^2^ = .57) with performance higher both post versus pre tDCS (t(14) = 3.35; p = .005) and post versus pre sham (t(14) = 3.64; p = .003), replicating the slight learning effect found in the accuracy results. No other main effect or interaction was significant, indicating tDCS did not modulate reaction times. Thus, overall tDCS had no impact in either VSTM accuracy or reaction times.

#### The impact of tDCS on confidence

The impact of tDCS on response confidence as a function of stimulus condition is shown in Figure 4B. An ANOVA with stimulus condition (BL, 0 deg difference, 10 deg difference, 40 deg difference), tDCS condition (anodal or sham) and session order (pre or post) revealed a significant main effect of stimulus condition (F(3,42) = 11,1; p<.001; partial η^2^ = .44) and a 2-way interaction between tDCS condition and session order (F(1,14) = 4.86; p = .045; partial η^2^ = .26). No other main effect or interaction was significant. Further analysis on the effect of stimulus condition indicated that, as in Experiment 1, confidence ratings were reduced (relative to the baseline) whenever the second memory cue was presented: *0 deg versus baseline*: t(14) = 4.1; p = .005; *10 deg versus baseline*: t(14) = 6.04; p<.001; *40 deg versus baseline*: t(14) = 4.49; p<.001).

To further investigate the interaction between tDCS condition (anodal/sham) and session order (pre/post) we carried out pairwise comparisons which revealed that confidence in the post-anodal condition was significantly lower relative to pre-anodal condition (t(14) = 2.57; p = .02). Confidence in the post-sham and pre-sham condition did not significantly differ (t(14) = .21; p = .84). Thus these analyses indicate that DLPFC tDCS induced a general reduction in the confidence ratings that was not modulated with the presence of the second memory cue or its orientation.

#### Summary of results of Experiment 2

In summary, the results of Experiment 2 can be summarized as: 1) anodal tDCS did not modulate VSTM accuracy (see Figure 4A); 2) anodal tDCS induced a general decrease in confidence rating that was not modulated by stimulus condition (see Figure 4B); 3) tDCS did not modulate participants' metacognitive sensitivity *per se*, (i.e. the correlation between VSTM accuracy and response confidence – this is reflected as no change in the slope of the psychometric function in Figure 3A); 4) tDCS modulated the bias of confidence ratings, reflected as a leftward shift in the psychometric function in Figure 3A.

## Discussion

In these experiments, we investigated the relationship between the objective and subjective components of VSTM and demonstrated that these aspects do not always go hand-in hand, neither at the behavioral nor at the cortical level. The main behavioral finding of both experiments was that VSTM accuracy and confidence are differentially affected by a visual stimulus presented during the delay period of an VSTM task. This is inconsistent with current models of working memory which posit that memory contents are highly accessible to conscious inspection [23], [24], as this would predict that VSTM and confidence should not be dissociated in this manner. With respect to existing theories, our results are in agreement with the *accessibility hypothesis* [22], according to which objective performance and subjective evaluation of one's own performance can be dissociable and are based, at least partially, on different sources of information [9], [17], [18], [19]. The present pattern of result is also consistent with those previously obtained for introspective aspects (subjective vividness) of VSTM content [25].

In Experiment 1, we behaviorally assessed how VSTM accuracy and confidence are affected by the presentation of a second memory cue during the maintenance period which either needed to be encoded into VSTM (active condition) or merely passively observed (passive condition). Our results show that the impact of the second memory cue on the accuracy of the first cue depended on their orientation similarity, with the effects becoming larger as the orientation difference was increased. This effect did not significantly vary across active and passive conditions (although a clear trend was present). For VSTM accuracy, the strongest reduction was observed when the orientations of the two memory cues differed by 40 degrees. This fits well with previous studies on memory masking investigating how visual distracters affect the accuracy of representations held in VSTM [76], [77]. In Magnussen's studies participants were asked to maintain in memory the spatial frequency of a memory cue and the disruptive effect of the visual distracter increased linearly with increasing spatial frequency difference between distracter and memory cue. The highest impairment was found at a difference of ±1 octave, corresponding to the width of spatial frequency channels reported in psychophysical studies [27], [28]. The present results are similar, as memory performance was reduced when the orientation difference between the two memory cues was increased. We found the largest impairment at 40 degrees, indicative of competition between orientation-selective channels, the width of which is believed to be in the range of 30-40 degrees (e.g. [27], [28], [29]). Simply increasing the memory load did not decrease VSTM performance; it was the similarity between the two memory items which determined VSTM accuracy. It is also important to note that the VSTM task required an explicit comparison between the orientations of the test stimuli and the memory cues and thus could not be accomplished by “passive” processes based on familiarity or recognition. The impact of the distracter cue on confidence ratings did not follow this pattern, as confidence ratings were reduced regardless of orientation difference between them (even though VSTM accuracy was not reduced when the two cues differed by 0 deg and 10 deg). Thus confidence ratings did not always reflect participants' VSTM accuracy. Taken together, these behavioral results contribute to the ongoing debate on the relationship between objective and subjective dimensions of memory, supporting the view that confidence and accuracy are not entirely based on the same source of information [5],[21].

In Experiment 2, we investigated the cortical basis of VSTM metamemory by assessing the role of the right dorsolateral prefrontal cortex in VSTM accuracy and confidence. Our results confirm the involvement of this brain region in confidence judgments, consistently with previous studies [21], [47], [48], [49], [78]. Specifically, we found that the application of tDCS over this area reduced confidence ratings, while leaving accuracy unaffected. Specifically, tDCS induced a *general reduction* in confidence ratings that was not modulated by the stimulus condition. In the psychometric function depicting the correlation between VSTM accuracy and confidence (Figure 3A), this was manifested as a leftward shift, with confidence ratings associated with a higher level of VSTM accuracy in the post-anodal tDCS condition relative to pre-anodal tDCS condition. The slope of this psychometric function was not affected, suggesting that tDCS did not modulate participants' metacognitive sensitivity *per se*, i.e. the correlation between accuracy and confidence (see Figure 3A). Statistically, this is indicated by the finding that the correlation between VSTM accuracy and confidence rating was very similar across the tDCS conditions. The simple explanation of this pattern of result is that tDCS had an effect on confidence bias, but the quality of the VSTM information underlying the confidence decision was unaffected. This is consistent with previous studies assessing the role of this region on monitoring processes with confidence judgments [47], [79]: in these studies dorsolateral prefrontal cortex showed a greater response for correct low-confidence judgments compared to correct high-confidence ones. This was explained in terms of low confidence judgments reflecting situations that are likely to require more monitoring of the retrieved information and furthermore they occur when the memory signal is close to decision criterion, requiring a greater evaluative component [47], [79]. It may be that the artificial enhancement of DLPFC activation induced by tDCS evoked the conditions in which low confidence judgments are made (i.e. higher activation level associated with lower confidence).

Several studies have successfully used tDCS to modulate working memory processes, with anodal stimulation of dorsolateral prefrontal cortex improving accuracy [65], [66], [67], [68], [80]; in this context, the lack of an effect here may seem surprising. One possibility for the lack of accuracy modulation is in terms of the easiness of the task. Baseline performance (i.e. the performance when the 2^nd^ memory cue is not presented) was high (0.84), and memory performance is not easily modulated by tDCS when this is the case [35]. The baseline level of confidence was in the middle of the 1–9 confidence scale (around 5.8), i.e. neither at floor or ceiling, and thus there may have been more scope for it to be modulated. An alternative explanation is that the maintenance of low-level orientation information relies more strongly on orientation channels in the early visual cortex rather than on DLPFC [77]. This would be consistent with a previous study using the same task and showing that TMS applied over V1 modulates VSTM accuracy [26]. The finding that accuracy was impaired by the second memory cue indicates that the task is susceptible to disruptive effects, and the nature of this impairment (with largest effect obtained with an orientation difference of 40 degrees) indicates that the memory performance did rely on orientation channels in the visual cortex (cf. [77]). Thus the actual memory maintenance, because it involves low-level visual features, may primarily involve the early visual areas.

Anodal tDCS, as used here, is believed to cause depolarization of neuronal membranes, resulting in an increased cortical excitability and facilitation of performance [51], [52], [81], [82]. Thus our results showing a reduction of confidence ratings might appear surprising. However the effects of anodal stimulation on cognitive functions are still controversial: for example, anodal stimulation of dorsolateral prefrontal cortex has been shown to *impair* performance in a categorization task [83] and slow down reaction times in a recognition paradigm [84]. Thus the view that anodal stimulation should lead to improvements of performance is too simplistic [85]. One possible explanation is that anodal tDCS adds noise to signal processing. In this view, the increased neural activity induced by anodal stimulation might lead to a decreased signal-to-noise ratio [86] whereas cathodal and sham tDCS might maintain the previous signal-to-noise ratio [87]. Consistent with this view, in our paradigm the increase of overall cortical excitability induced by anodal tDCS might have elevated the activation state of all the neurons, adding noise to the neuronal representations in DLPFC on which the metacognitive judgment is based.

As VSTM accuracy was not affected by tDCS, the reduction in confidence rating is not simply a byproduct of a worsening memory performance. Similarly, in Experiment 1, the presentation of second cue reduced confidence ratings in specific conditions without affecting accuracy. Changes in discrimination performance between conditions can complicate the interpretation of metacognitive sensitivity, as it can be difficult to determine whether changes in metacognition are due to the experimental manipulation affecting metacognitive abilities, or whether the worsening of task performance changes the coupling between accuracy and confidence (see [88]). This problem is not present here due to the lack of accuracy effects by tDCS or the second cue in specific conditions.

At first sight, the effects of tDCS on confidence ratings fit well with several studies implicating this region in metacognition processes [21], [46], [47], [48] as well as in visual consciousness in general [2], [89]. For example, bilateral application of TMS over the DLPFC has been shown to reduce metacognitive abilities in a visual detection task [90]. However, in the study by Rounis et al. [90] it was not the bias but rather the metacognitive sensitivity (i.e. the correlation between accuracy and confidence) that was reduced, whereas in the present study this correlation was unaffected. One important difference between our experiment and the study by Rounis et al. [90] was that here stimulation was unilateral; it may be that bilateral disruption of the PFC is required for metacognitive sensitivity to be disrupted. This could reflect the importance of both the left and right DLPFC in metacognition, with disruption of only one hemisphere being insufficient to modulate metacognitive sensitivity, due to the ability of the non-stimulated hemisphere to function normally. Furthermore, TMS is likely to be a much more robust technique for modulating cognitive performance in comparison to tDCS. It is important to stress however that in the present study, tDCS did modulate metacognition (i.e. we did no obtain a null effect), but only with respect to metacognitive bias.

To date, the role of the DLPFC in memory monitoring has been mostly investigated in relation to episodic memory; our results extend these findings to visual short-term memory. Furthermore, our results demonstrate that participants do not always have an accurate insight to their WM performance, indicating that our experience of memory processes may not always reflect the accuracy of the underlying memory representation. In other words, subjective and objective components of VSTM are dissociable processes (see also [25]).
